# Effects of Human Activities on Evapotranspiration and Its Components in Arid Areas

**DOI:** 10.3390/ijerph20042795

**Published:** 2023-02-04

**Authors:** Yunfei Liu, Dongwei Gui, Changjun Yin, Lei Zhang, Dongping Xue, Yi Liu, Zeeshan Ahmed, Fanjiang Zeng

**Affiliations:** 1 State Key Laboratory of Desert and Oasis Ecology, Xinjiang Institute of Ecology and Geography, Chinese Academy of Sciences, Urumqi 830011, China; 2 Cele National Station of Observation and Research for Desert-Grassland Ecosystems, Cele 848300, China; 3 College of Resources and Environment, University of Chinese Academy of Sciences, College of Resources and Environment, Beijing 100049, China

**Keywords:** arid areas, evapotranspiration, human activities, different land-use types, hydrological process

## Abstract

With the increasing impact of human activities on the environment, evapotranspiration (ET) has changed in arid areas, which further affects the water resources availability in the region. Therefore, understanding the impact of human activities on ET and its components is helpful to the management of water resources in arid areas. This study verified the accuracy of Fisher’s model (PT-JPL model) for ET estimation in southern Xinjiang, China by using the evaporation complementarity theory dataset (AET dataset). The ET and the evapotranspiration components (T:E) of six land-use types were estimated in southern Xinjiang from 1982 to 2015, and the impact of human activities on ET was analyzed. In addition, the impact of four environmental factors (temperature (Temp), net radiation (Rn), relative humidity (RH), and NDVI) on ET were evaluated. The results showed that the calculated ET values of the PT-JPL model were close to the ET values of the AET dataset. The correlation coefficient (R^2^) was more than 0.8, and the NSE was close to 1. In grassland, water area, urban industrial and mining land, forest land, and cultivated land, the ET values were high, and in unused land types, the ET values were the lowest. The T:E values varied greatly in urban industrial and mining land, forest land, and cultivated land, which was due to the intensification of human activities, and the values were close to 1 in summer in recent years. Among the four environmental factors, temperature largely influenced the monthly ET. These findings suggest that human activities have significantly reduced soil evaporation and improved water use efficiency. The impact of human activities on environmental factors has caused changes in ET and its components, and appropriate oasis expansion is more conducive to regional sustainable development.

## 1. Introduction

ET is water from different underlying surfaces releasing into the atmosphere through the effect of various ecological, climatic, and environmental factors [1,2]. It is an important factor in evaluating the eco-environmental system stability and also plays an important role in water resource management and decision-making [3,4]. In arid areas, the ET monitoring is of great significance to the utilization of water resources, oasis expansion, and regional sustainable development [5,6,7]. ET in arid areas is greatly affected by climate change. Meanwhile, human activities that include agricultural planting and artificial shelter forest construction changed the underlying surface environment; thus, ET was also significantly affected by human activities. However, due to the limitation of data and methods, how the variation trend of ET and T:E is affected by human activities in arid areas and the main driving factors of the variation trend still need to be studied.

Evapotranspiration components include soil evaporation (E), vegetation transpiration (T), and canopy interception [8,9]. Because of the small amount of precipitation in the arid areas, canopy interception can be ignored in the calculation. E is closely related to the underlying surface soil moisture, which indicates the soil moisture loss. In the case of little change in soil moisture, the changes of E reflect the changes of vegetation water use efficiency [10]. T reflects the changes of plant growth density and land-use type, which is related to the species, density, and growth environment of the plant. In oasis area, T is greatly affected by soil utilization intensity and plant growth state [11]. The accurate calculation of T:E is conducive to understanding the impact of regional human activities and climate change on hydrological processes [12,13].

At present, the calculation of T:E is very difficult in arid areas. In recent years, many research achievements have been made in estimating T:E in arid areas. S. Kodur [14] used the APSIM farming system model to improve the soil evaporation prediction for different soil types in arid areas. The prediction accuracy of crop yield was improved; Wang et al. [15] studied the evapotranspiration components in desert and alpine ecosystems in the arid area of northwest China, which provided a basis for the analysis of underlying surface water use efficiency change. In another study, N.C. Mbangiwa et al. [16] simulated and predicted the evapotranspiration of dry land soybean in KwaZulu Natal, South Africa, which was useful for crop water management. Italo Sampaio Rodrigues et al. [17] analyzed the impact of dry land vegetation on reservoir evaporation in Brazil and quantified the uncertain factors of reservoir evaporation. Many studies have focused on small-scale areas, relying on a large amount of flux observations and meteorological station data to simulate and calculate ET and its components. Due to the vast area and lack of data, more appropriate methods are needed for the ET estimation and component segmentation of ET in arid areas [18,19], but there are few studies on the ET segmentation of a large-scale complex underlying surface in arid areas.

South of Xinjiang, China, there are typical hyper-arid areas. Due to the special natural and geographical environment of this region, it is very difficult to measure ET [20]. With the development of remote sensing technology, it provides conditions for ET calculation and segmentation in the region. Therefore, the objectives of this study are (1) to calculate the ET and its components in southern Xinjiang; (2) to analyze the variation trend and change driving factors of ET and its components in six land-use types that include cultivated land, urban industrial and mining land, forest land (greatly affected by human activities), grassland, water area (less affected by human activities), and unused land (not affected by human activities); (3) to study the effects of human activities on ET and its components. This study is helpful to understand the hydrological process change in arid areas with climate change and to provide reference for the study of the interaction among ET, environmental factors, and human activities. At the same time, the study can guide the formulation of the water resource policy in arid areas and provide basis for the rational development and utilization of water resources. The results can provide support for protecting the ecological environment and maintaining the sustainable development of oasis in arid areas.

## 2. Materials and Methods

### 2.1. Study Area

**Figure 1:**
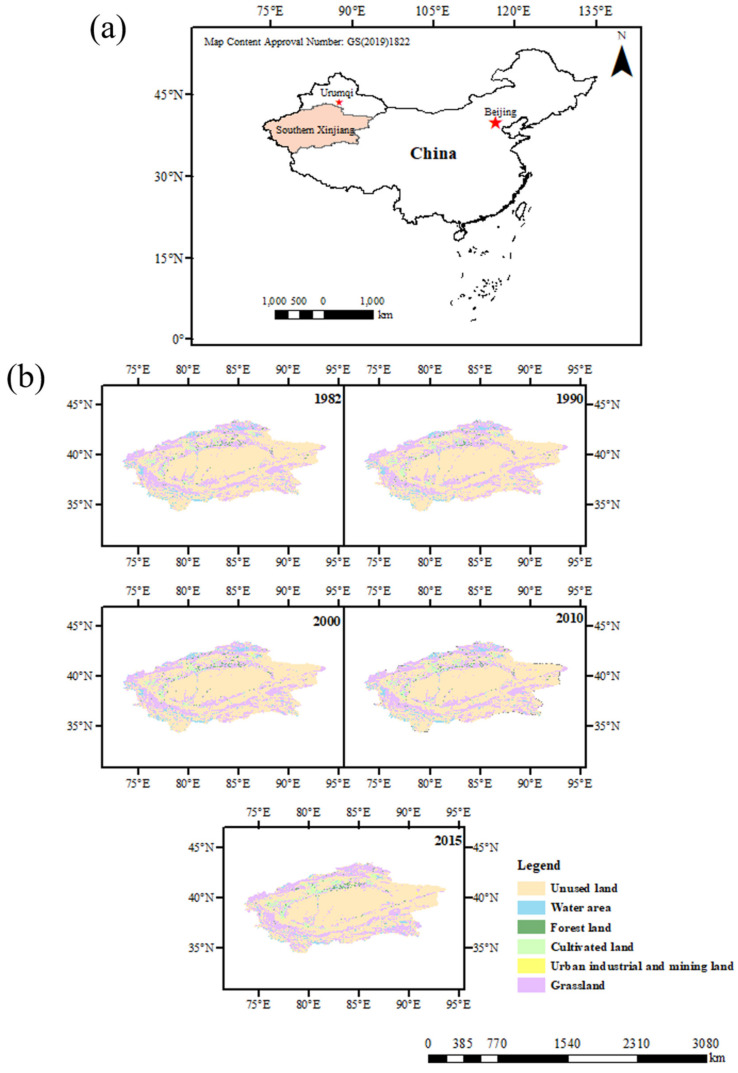
(**a**) Location map of the study area. (**b**) Land-use type changes in southern Xinjiang from 1982 to 2015.

Southern Xinjiang is located in the inland at mid latitude (Figure 1). It is a hyper-arid area and is characterized by dry climate, low rainfall, large potential evapotranspiration, and limited water resources [21]. The social production activities in this region are mainly concentrated in the low altitude plain oasis area [22]. In recent years, with the intensification of human activities, the cultivated land area has expanded by 17,071 km^2^, and the water area and grassland have decreased by 12,447 km^2^ and 14,321 km^2^, respectively (Table 1). The contradiction between limited water resources and human activities has intensified [23]. In this study, the land-use types in southern Xinjiang were divided into six categories, including cultivated land, forest land, grassland, water area, unused land, and urban industrial and mining land.

### 2.2. PT-JPL Model

The PT-JPL model is based on the Priestley–Taylor model and satellite remote sensing data. In order to simplify the large-scale actual evapotranspiration calculation, the model reduces the difficulty of parameter acquisition [24]. The PT-JPL model divides the ET into three parts: canopy interception evaporation, soil evapotranspiration, and plant transpiration, as shown in the following equation:

(1) $${\mathrm{ET}=\mathrm{ET}}_{i}{+\mathrm{ET}}_{c}{+\mathrm{ET}}_{s}$$

In Equation (1), ET is the total actual evapotranspiration (mm); ET_i_ is canopy interception evaporation (mm); ET_c_ is plant transpiration (mm); ET_s_ is soil evaporation (mm).

According to Equation (1), the three components of evapotranspiration are calculated by using the PT-JPL model as follows:

(2) $${\mathrm{ET}}_{c}=(1\;-f_{\mathrm{wet}}{)f}_{g}f_{T}f_{M}\alpha \frac{\Delta }{\Delta +\gamma }R_{\mathrm{nc}}$$

(3) $${\mathrm{ET}}_{s}{=(f}_{\mathrm{wet}}{+f}_{\mathrm{SM}}(1\;-{\;f}_{\mathrm{wet}}))\alpha \frac{\Delta }{\Delta +\gamma }{(R}_{\mathrm{ns}}-\;G)$$

(4) $${\mathrm{ET}}_{i}=f_{\mathrm{wet}}\alpha \frac{\Delta }{\Delta +\gamma }R_{\mathrm{nc}}$$

where *f_wet_* is the surface relative humidity (%); *f_g_* is the green canopy fraction, dimensionless; *f_T_* is the vegetation temperature limit index, dimensionless; *f_M_* is the plant water restriction index, dimensionless; *f_SM_* is the limiting factor of soil water, dimensionless; *α* is the coefficient in the Priestley–Taylor formula; Δ is the slope of temperature saturated water pressure (kPa·°C^−1^); γ is the dry and wet meter constant (kPa·°C^−1^); R_nc_ is Net Canopy radiation (MJ·m^−2^); R_ns_ is the net radiation of soil (MJ·m^−2^); *G* is soil heat flux (MJ·m^−2^). In the formula, R_ns_ is the comprehensive leaf area index (LAI), net radiation (Rn), and radiation correction coefficient of the observation area. The value and detailed calculation process of all variables can be obtained from the model introduction by Fisher [24].

In the PT-JPL model, the numbers of parameters are reduced in calculations so that all parameters are obtained from satellite data. Fisher et al. (2008) compared the model calculation results with the ET monitoring results, and the R^2^ was more than 0.9.

### 2.3. Data

The AET dataset is the shared data developed by the department of Geosciences, Tsinghua University. In the dataset, the ET is estimated by using the evaporation complementarity theory. The AET dataset adopts strict data quality control, the unified fusion method of station data, satellite data, and reanalysis data, and it has higher accuracy than the GLDAS dataset that is widely used in the world [25].

The meteorological data, including Rn, RH, Temp, atmospheric pressure, etc., was obtained from the China meteorological forcing dataset (CMFD), with the spatial resolution of 0.1 degrees and the temporal resolution of 3 h (https://data.tpdc.ac.cn/zh-hans/data/8028b944-daaa-4511-8769-965612652c49/, accessed on 17 August 2021). The NDVI and SEVI data used in this study were calculated and analyzed by geospatial data cloud (http://www.gscloud.cn/, accessed on 20 April 2022). The land-use and land-cover change in southern Xinjiang was obtained from the Chinese Academy of Sciences LUCC data.

### 2.4. Model Test Method

In order to analyze the prediction performance of the model, the commonly used error index system was used to evaluate the results. The main evaluation indexes include decisive coefficient (R^2^), Nash Sutcliffe Nash efficiency coefficient (NSE), and root mean square error (RMSE). The calculation method is as follows:

(5) $$\mathrm{RMSE}=\sqrt{\frac{1}{T}\sum_{t=1}^{T}{{(\mathrm{ET}}_{\mathrm{PT}\;-\;\mathrm{JPL}}^{t}-{\mathrm{ET}}_{\mathrm{AET}}^{t})}^{2}}$$

(6) $$\mathrm{NSE}=1\;-\frac{\sum_{t=1}^{T}{{(\mathrm{ET}}_{\mathrm{PT}-\mathrm{JPL}\;}^{t}-{\;\mathrm{ET}}_{\mathrm{AET}}^{t})}^{2}}{\sum_{t=1}^{T}{{(\mathrm{ET}}_{\mathrm{AET}}^{t}-\bar{{\;\mathrm{ET}}_{\mathrm{AET}}})}^{2}}$$

(7) $$R^{2}={\left(\frac{\Sigma \left(x-\bar{x}\right)\left(y-\bar{y}\right)}{\sqrt{\Sigma {\left(x-\bar{x}\right)}^{2}\Sigma {\left(y-\bar{y}\right)}^{2}}}\right)}^{2}$$

In Equation (5), *T* is the time series (year); ET_PT-JPL_ is the ET value calculated by the PT-JPL model (mm); *ET_AET_* is the ET value in the AET dataset (mm); $\bar{{\mathrm{ET}}_{\mathrm{AET}}}$ is the average value (mm) of the AET dataset. In Equation (6), the NSE range is negative infinity to 1. When the NSE is closer to 1, it means that the calculation result of the PT-JPL model is close to the AET dataset, and when the NSE is closer to 0, it means that the calculation result of the PT-JPL model is close to the ET mean value of AET dataset, but the error is large. When the NSE value is much less than 0, it means that the calculation result of the PT-JPL model is poor and the result is unreliable. In Equation (7), *x* is the value in the first group of samples, $\bar{x}$ is the average value of the first group of samples, *y* is the median value of the second group of samples, and $\bar{y}$ is the average value of the second group of samples.

The Mann-Kendall test (M-K) was used for environmental factor change analysis. The data analysis and processing tools used in this paper mainly include ArcGIS10.6, R language, and Excel.

## 3. Results

### 3.1. Applicability Analysis of PT-JPL Model in Southern Xinjiang

**Figure 2:**
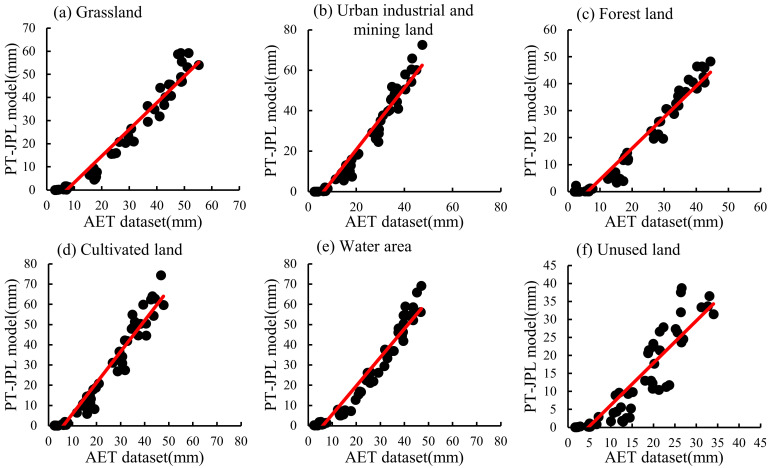
Comparison of the evapotranspiration of different land-use types between the AET dataset and the PT-JPL model. (**a**–**f**) describe the comparison of evapotranspiration in grassland, urban industrial and mining land, forest land, cultivated land, water area, and unused land, respectively.

The accuracy and applicability of the AET dataset in China was proven by Sun et al. (2021). In this study, the AET dataset was used as the actual evapotranspiration value to compare with the calculation results by the PT-JPL model in different land-use types in southern Xinjiang during 1982~2015 (Figure 2). The results of the PT-JPL model and the AET dataset were relatively close, and the values were concentrated near the trend line in six land-use types. The R^2^ was mostly above 0.9 in different years, and the R^2^ of unused land, water area, forest land, cultivated land, urban industrial and mining land, and grassland were 0.84, 0.95, 0.95, 0.95, 0.95, and 0.95, respectively (Table 2). NSE was greater than 0 and close to 1, and the RMSE value was low, indicating that the results of the PT-JPL model were accurate in southern Xinjiang (Table 2).

**Figure 3:**
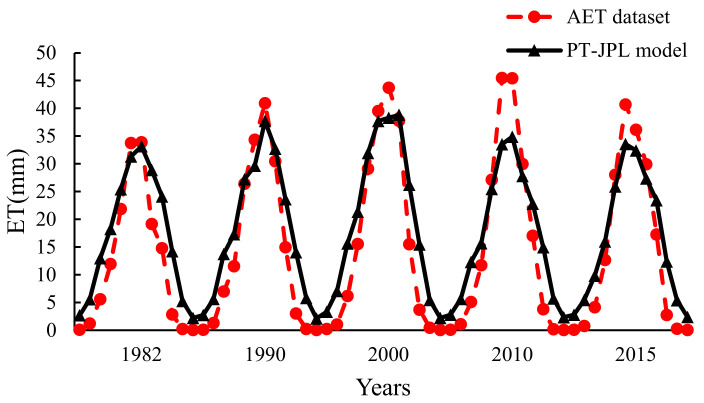
Comparison of annual evapotranspiration data of the AET dataset and the PT-JPL model in southern Xinjiang from 1982 to 2015.

The calculation results of the PT-JPL model in southern Xinjiang were close to those of the AET dataset from 1982 to 2015 (Figure 3). The ET values were about 40 mm in June and July every year, which were the highest values. In January and December, ET values were the lowest, which was close to 0 mm. The annual ET value in southern Xinjiang showed a weak upward trend from 1982 to 2015. The ET values of the PT-JPL model in different months were significantly different, while the ET values of the AET dataset had almost no change from November to the next year in February, and the values were low. Since 2000, the peak value of ET in the AET dataset was higher than that in the PT-JPL model (Figure 3).

The results between the AET dataset and the PT-JPL model were close in different land-use types. However, in unused land, forest land, and grassland, the values of the PT-JPL model were slightly higher than those of the AET dataset, and the trend of ET was opposite in urban industrial and mining land and cultivated land between them. The ET values of the PT-JPL model in the water area were close to those of the AET dataset. In general, the inter-annual variation of ET values of the PT-JPL model were in the range of 196.84~243.5 mm, while ET values of the AET dataset were in the range of 145.40~192.68 mm. Both of them showed an upward trend from 1982 to 2000 and decreased slightly after 2000 (Table 3).

In conclusion, compared to the AET dataset, the error of the PT-JPL model was low, and the calculation results of the model were reliable. Therefore, the PT-JPL model was suitable for the calculation of ET in southern Xinjiang and had high accuracy.

### 3.2. Evapotranspiration and its Component Change Characteristics of Different Land-Use Types in Southern Xinjiang

The PT-JPL model was applicable in southern Xinjiang; thus, this model could be used to divide ET into soil evaporation, vegetation transpiration, and canopy interception evapotranspiration. By using the PT-JPL model for calculations, the ET of different land-use types in southern Xinjiang in 1982, 1990, 2000, 2010, and 2015 was divided into three parts. The water area E is composed of water evaporation and soil evaporation near the water body, and T is composed of vegetation transpiration near the water body.

**Figure 4:**
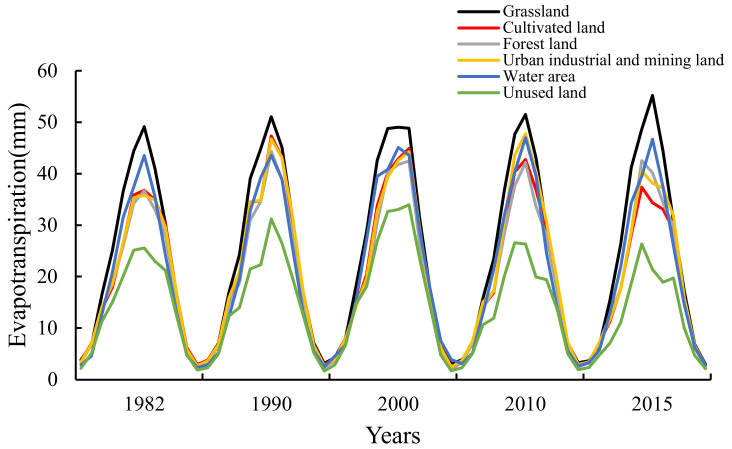
Change trend of monthly evapotranspiration data of different land-use types in southern Xinjiang.

In southern Xinjiang, the evapotranspiration change of different land-use types was the same from 1982 to 2015 (Figure 4). The maximum ET value appeared in June or July every year, and the lowest value appeared in January or December. The ET values of different land-use types were close at a low value, but there were differences at the peak value. The peak value of unused land was about 25 mm, and that of grassland was about 50 mm. The ET variation trend of forest land, urban industrial and mining land, and cultivated land was similar, and the peak value changed in different years. The ET peak value of water body was stable at about 45 mm.

**Figure 5:**
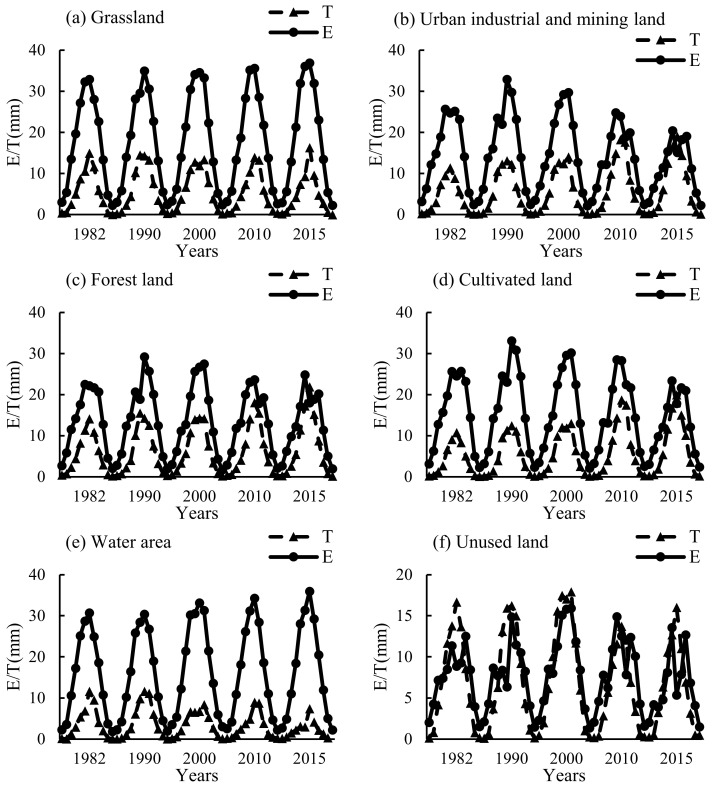
Comparison of monthly data of vegetation transpiration and soil evaporation in different land-use types from 1982 to 2015. (**a–f**) describe the variation trend of E/T in grassland, urban industrial and mining land, forest land, cultivated land, water area, and unused land, respectively.

The proportion and variation trend of E/T were different in diverse land-use types. On the whole, the changes of E/T values were affected by vegetation growth status and meteorological factors, and the E/T trends were consistent with the ET trend (Figure 4 and Figure 5). The trends of E/T values in grassland and water area from 1982 to 2015 were stable, and ET values changed little every year. The ET of water area showed a slight upward trend. After 1990, in industrial and mining land, forest land, and cultivated land, the E values decreased, but the T values increased gradually, and the peak values and the variation trend of E/T were close in 2015. In unused land, the E/T values were similar. The T values were slightly greater than the E values from June to July, and the E values fluctuated greatly from May to August.

In different seasons, the T:E value changes in water area and grassland were gentle, with the maximum value in summer, close in autumn and spring, and the minimum in winter. The maximum T:E values appeared in the grassland and water area in 1990, which were 0.38 and 0.45, respectively, and the T values were much lower than the E values. In urban industrial and mining land, forest land, and cultivated land, the T:E values were in the following order, summer > spring > autumn > winter, and they showed a gradual increasing trend. The maximum values appeared in the 2015 summer, which were 0.91, 0.95, and 0.84, respectively. Each year, T:E values in unused land were larger than those in other land-use types. Except for 0.58 in 2010, the ratio was about 0.8 in other years, and T values were greater than E values in spring and summer (Table 4).

### 3.3. Variation Trend of Environmental Factors and the Impact on ET of Different Land-Use Types in the Southern Xinjiang

**Figure 6:**
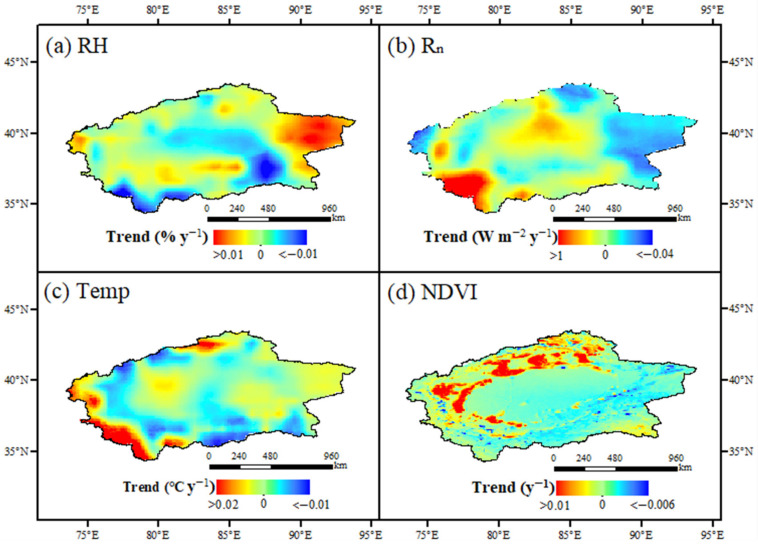
Variation trend of Temp, Rn, RH, and the NDVI in southern Xinjiang from 1982 to 2015. (**a–d**) describe the variation trend of relative humidity, net radiation, temperature and NDVI, respectively.

**Figure 7:**
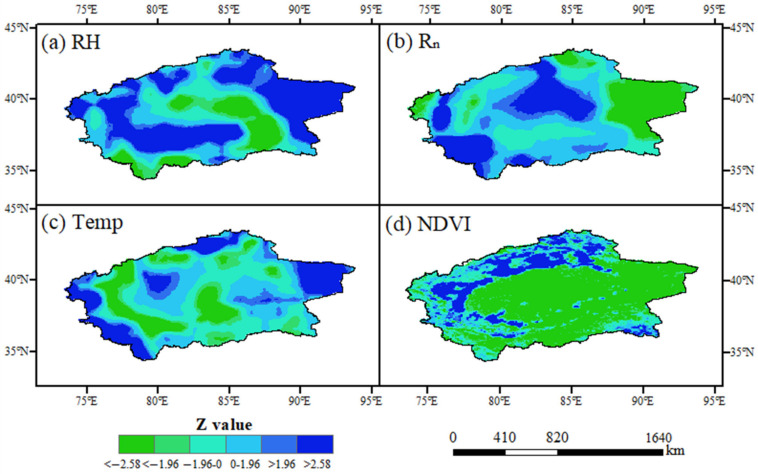
The M-K Test Z value of Temp, Rn, RH, and the NDVI in southern Xinjiang from 1982 to 2015. (**a–d**) describe the Z value of relative humidity, net radiation, temperature and NDVI, respectively.

The change of climate factors has a great impact on evapotranspiration. Therefore, it is necessary to analyze the trend of environmental factors for understanding the fluctuations in evapotranspiration. In this study, the M-K test was used to analyze the variation trends of relative humidity (RH), net radiation (Rn), temperature (Temp), and the vegetation normalization index (NDVI) in southern Xinjiang from 1982 to 2015. The results revealed that the RH increased about 0.01%/y in the eastern desert area and decreased about 0.01%/y in the mountainous area of the southeast and southwest. Overall, the RH in most areas was a significant increasing trend, whereas the RH in the central desert area decreased significantly (Figure 6a and Figure 7a). Rn increased significantly in most areas except the east, especially in the mountainous grassland in the southwest, and its increasing rate reached about 1 W/m^2^·y. Temp increased significantly in the eastern desert, western mountainous area, and northern grassland of southern Xinjiang and decreased in other areas, but the change rate was very slow (Figure 6c and Figure 7c). The NDVI increased significantly in the oasis area and the grassland area in the west, north, and southeast at a rate of about 0.01/y, while it decreased in the desert area in the east, central part, and most areas of mountain grassland in the south, but the decrease rate was slow (Figure 6d and Figure 7d).

**Figure 8:**
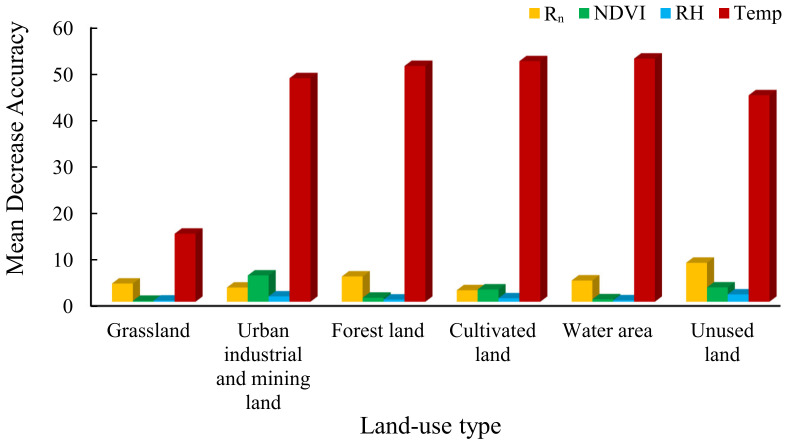
Effects of meteorological factors on the evapotranspiration of different land-use types.

We used four environmental factors as input parameters, and monthly ET values were used as output. The mean decrease accuracy value was used to determine the importance of each factor by the Random forest model. Figure 8 showed the statistical results of ET values for six land-use types, as affected by four meteorological environmental factors. The mean decrease accuracy value of parameters was high, indicating that the influence of parameters on ET was great. In general, temperature had the greatest influence on the change of monthly ET values. NDVI values were lower than the net radiation, except for cultivated land and urban industrial and mining land. Relative humidity had little effect on ET. The monthly ET data used may be the reason why Temp became the main factor affecting ET changes. Climate change is the main driving factor of monthly ET change. The impact of human activities on ET needs to be assessed on a longer time scale.

## 4. Discussion

### 4.1. Effects of Six Land-Use Types on ET Calculation in Southern Xinjiang

In this study, land-use types in southern Xinjiang were divided into six categories, in which grassland was mainly distributed in the middle altitude areas of the Kunlun Mountain and Tianshan Mountain, near the water area in the desert and around the oasis with more precipitation (Figure 1). It was less affected by moisture and temperature; thus, the ET was high [26,27] (Figure 4). Cultivated land was mainly distributed in the plain desert area near the river (Figure 1); therefore, it mainly depended on artificial irrigation and was greatly affected by agricultural activities, plant growth status, and environmental factors, and the ET was higher than other areas from May to September [28] (Figure 4 and Figure 5). Urban industrial and mining land and forest land were mainly distributed in the oasis area near farmland with a small area (Figure 1) and were largely affected by human activities. In addition, due to the data resolution, it might be affected by farmland data; thus, the ET values were close to farmland (Table 1, Figure 4). The unused land was mainly in the Taklimakan Desert area in southern Xinjiang and the high-altitude area (Figure 1), which was greatly affected by the underlying surface moisture or temperature; thus, the ET was low [29] (Figure 4). The water area comprised of rivers and lakes. Rivers were mainly distributed in low-altitude areas, while lakes were mainly distributed in medium- and high-altitude areas, and a few were distributed near an oasis (Figure 1). Although the ET of water area was less limited by water, the low temperature of plateau lakes limited the ET. Thus, the water area ET values were lower than those of grassland areas and close to those of oasis areas [30]. Human activities are the main reason for the area change of different land-use types. The underlying surface environment changes affect the regional hydrological process and water availability. Therefore, it is necessary to pay attention to the changes of ET and its components in different land-use types to ensure that human activities do not have adverse effects on the environment [31,32].

### 4.2. Changes of Human Activities on Environmental Factors

Human activities have a significant impact on the climate and environment in southern Xinjiang, China [33]. Human activities in southern Xinjiang are mainly concentrated in oases, which are agricultural planting. With the oasis expansion, human activities greatly increased the agricultural planting area [34]. Cultivated land increased the amount of water consumption in oases, which made the ET show an obvious increasing trend since 1982. This led to an increase in the moisture content of the underlying surface entering the atmosphere; thus, the RH rose [35]. In addition, the increase of cultivated land increased the vegetation coverage on the underlying surface. The transpiration of vegetation caused the decrease of regional Temp. The increase of the NDVI also enhanced the surface radiation reflectivity and reduced the value of Rn [36] (Figure 6 and Figure 7). The changes of these environmental factors have a great impact on the regional ET [37].

### 4.3. Changes of ET in Different Land-Use Types Caused by Human Activities

The change of land-use type was usually accompanied by human activities. The unused land in southern Xinjiang was mainly affected by the regional water factor due to less human interventions. Although the area of water and grassland decreased a lot, the ET in the area was less affected by water. Therefore, the changes of ET and its components were small. The areas mostly affected by human activities were urban industrial and mining land, forest land, and cultivated land. From 1982 to 2015, the cultivated land of the oasis in southern Xinjiang expanded by nearly 70%. With the intensification of human planting activities, ET values of urban industrial and mining land, forest land, and cultivated land changed largely (Table 3), and the T:E ratio showed an increasing trend since 1982. It reflected the improvement in water use efficiency of oasis planting, which was due to the adjustment of planting structure and the change of irrigation mode [38]. The promotion of intercropping mode, drip irrigation, and soil film-mulch planting greatly reduced soil evaporation and increased the proportion of crop transpiration in ET [39]. Moreover, the increase in oasis area indicated the microclimate effect of oases, which reduced the temperature and saturated water vapor pressure difference in the oasis area (Figure 6a,c and Figure 7a,c), and the moisture on the underlying surface was limited and decreased the ET [40]. On the other hand, the increase of the oasis T:E ratio also showed an increase in plant density and land-use intensity in the oasis. In the long run, the sustainability of oasis expansion for regional development needs further investigation [41].

## 5. Conclusions

The accurate measurement of ET and its components on a large-scale area is a challenging task in arid regions, owing to meteorological data limitation. In this study, the applicability of the PT-JPL model in an arid area was verified; the variation trend of ET and its components was analyzed in six different land-use types. The calculation of ET values in southern Xinjiang by the PT-JPL model remained close to the ET values in the AET dataset. R^2^ was more than 0.8, and the NSE was greater than 0, close to 1, indicating that the calculation results of the model had high reliability and applicability in southern Xinjiang. Among the different land-use types, grassland area, water area, urban industrial and mining land, forest land, and cultivated land revealed higher ET values, while unused land showed the lowest ET value. Unused land showed the highest T:E values, and the T:E change in grassland and water area was slow from 1982 to 2015. The T:E value varied greatly in urban industrial and mining land, forest land, and cultivated land mainly, owing to the intensification of human activities, and it was close to 1 in summer in recent years. Temperature had the greatest impact on the change in monthly ET value, and it was followed by Rn and the NDVI, while RH did not show a significant impact. The findings suggest that the PT-JPL model has few input parameters that can be obtained from satellite remote sensing inversion data, and it provides a method for the study of ET estimation and ET segmentation in arid areas. Human activities have significantly reduced soil evaporation and improved water use efficiency. The impact of human activities on environmental factors has caused changes in ET and its components, and appropriate oasis expansion is more conducive to regional sustainable development. In future studies, the appropriate scale of oasis and artificial protective forest expansion in the desert still needs to be explored.

## Figures and Tables

**Table 1:** Area changes of different land-use types in southern Xinjiang from 1982 to 2015.

Land-Use Type	Area in 1982 (km^2^)	Area in 1990 (km^2^)	Area in 2000 (km^2^)	Area in 2010 (km^2^)	Area in 2015 (km^2^)	Area Change from 1982 to 2015 (km^2^)
Cultivated land	25,163	24,479	26,908	31,659	42,234	17,071
Forest land	12,933	11,017	14,153	13,510	12,847	−86
Grassland	286,686	287,894	281,588	279,093	272,365	−14,321
Water area	40,441	39,782	41,210	40,940	27,994	−12,447
Urban industrial and mining land	1423	1601	1437	1698	3060	1637
Unused land	669,610	671,483	670,960	669,356	677,756	8146

**Table 2:** Error statistics of the PT-JPL model.

Land-Use Type		Year	1982	1990	2000	2010	2015	All
	Index							
Unused land	R^2^		0.76	0.87	0.89	0.83	0.87	0.84
	NSE		0.56	0.78	0.79	0.76	0.83	0.87
	RMSE (mm·mon^−1^)		6.56	5.47	6.34	6.78	4.70	6.02
Water area	R^2^		0.96	0.96	0.94	0.96	0.97	0.95
	NSE		0.92	0.91	0.84	0.83	0.89	0.93
	RMSE (mm·mon^−1^)		5.22	5.93	9.78	10.26	6.85	7.88
Forest land	R^2^		0.95	0.96	0.96	0.95	0.95	0.95
	NSE		0.83	0.89	0.91	0.86	0.84	0.94
	RMSE (mm·mon^−1^)		5.76	5.64	5.66	6.00	5.95	5.80
Cultivated land	R^2^		0.97	0.96	0.97	0.96	0.92	0.95
	NSE		0.83	0.76	0.81	0.89	0.89	0.92
	RMSE (mm·mon^−1^)		8.04	12.94	10.90	7.12	6.31	9.40
Urban industrial and mining land	R^2^		0.97	0.96	0.97	0.96	0.93	0.95
	NSE		0.85	0.79	0.84	0.88	0.89	0.92
	RMSE (mm·mon^−1^)		7.54	12.15	9.56	6.76	5.91	8.68
Grassland	R^2^		0.95	0.96	0.95	0.95	0.96	0.95
	NSE		0.83	0.88	0.87	0.89	0.92	0.94
	RMSE (mm·mon^−1^)		7.14	6.63	7.27	7.20	6.49	6.96

**Table 3:** Annual evapotranspiration data of different land-use types in the AET dataset and the PT-JPL model.

Land-Use Type		Year		1982	1990	2000	2010	2015
	ET (mm)							
Unused land	PT-JPL model			168.34	174.41	213.38	160.80	147.10
	AET dataset			110.09	135.07	160.26	152.27	127.32
Water area	PT-JPL model			233.73	241.30	280.74	245.25	251.49
	AET dataset			217.43	229.20	287.06	278.54	247.79
Forest land	PT-JPL model			224.66	244.73	247.84	234.92	239.76
	AET dataset			168.67	203.55	216.32	186.72	181.09
Cultivated land	PT-JPL model			231.06	264.78	258.79	262.77	241.69
	AET dataset			257.51	332.21	299.33	266.02	221.47
Urban industrial and mining land	PT-JPL model			232.65	264.69	262.34	245.64	225.12
	AET dataset			246.77	319.02	286.81	241.25	204.07
Grassland	PT-JPL model			279.93	290.80	306.98	283.44	299.29
	AET dataset			206.33	227.49	244.17	247.83	263.44
Southern Xinjiang	PT-JPL model			204.08	212.42	243.50	203.88	196.84
	AET dataset			145.40	170.21	192.68	186.98	172.74

**Table 4:** Seasonal and inter-annual variation of T:E values of different land-use types.

Year	Season	Unused Land	Water Area	Forest Land	Cultivated Land	Urban Industrial and Mining Land	Grassland
1982	Spring	1.00	0.17	0.33	0.20	0.23	0.26
	Summer	1.52	0.33	0.55	0.37	0.39	0.40
	Autumn	0.47	0.16	0.22	0.14	0.17	0.20
	Winter	0.13	0.03	0.13	0.06	0.07	0.09
	Annual average	0.78	0.17	0.31	0.19	0.22	0.24
1990	Spring	0.94	0.16	0.31	0.24	0.28	0.25
	Summer	1.64	0.38	0.60	0.41	0.46	0.45
	Autumn	0.56	0.24	0.27	0.18	0.21	0.27
	Winter	0.10	0.03	0.13	0.06	0.07	0.11
	Annual average	0.81	0.21	0.33	0.22	0.26	0.27
2000	Spring	1.12	0.20	0.43	0.31	0.35	0.32
	Summer	1.12	0.23	0.53	0.43	0.46	0.37
	Autumn	0.66	0.19	0.30	0.20	0.22	0.28
	Winter	0.27	0.06	0.17	0.09	0.10	0.15
	Annual average	0.79	0.17	0.36	0.26	0.28	0.28
2010	Spring	0.70	0.13	0.29	0.28	0.35	0.22
	Summer	1.14	0.24	0.75	0.65	0.77	0.38
	Autumn	0.35	0.14	0.29	0.27	0.30	0.21
	Winter	0.12	0.09	0.12	0.08	0.09	0.12
	Annual average	0.58	0.15	0.36	0.32	0.38	0.23
2015	Spring	1.16	0.12	0.46	0.44	0.52	0.20
	Summer	1.78	0.15	0.91	0.84	0.95	0.33
	Autumn	0.38	0.09	0.30	0.30	0.31	0.17
	Winter	0.19	0.05	0.11	0.08	0.08	0.07
	Annual average	0.88	0.10	0.44	0.42	0.47	0.19

## Data Availability

The datasets used and/or analyzed during the current study are available from the corresponding author on reasonable request.
